# Inhibition of retinal neovascularization by Dendrobium polysaccharides: a review

**DOI:** 10.3389/fphar.2025.1584553

**Published:** 2025-06-30

**Authors:** Dan Liu, Xingwang Chen, Shanjun Cai

**Affiliations:** ^1^ Ophthalmology Department, Affiliated Hospital of Zunyi Medical University, Zunyi, Guizhou, China; ^2^ Guizhou Eye Hospital, Zunyi, Guizhou, China; ^3^ Guizhou Branch Center of National Clinical Research Center for Ophthalmology, Zunyi, Guizhou, China; ^4^ Key Laboratory of Eye Diseases, High Education Institution in Guizhou Province, Zunyi, Guizhou, China

**Keywords:** dendrobium polysaccharides, retinal neovascularization, vascular endothelial growth factor, antioxidant, anti-inflammatory

## Abstract

Retinal neovascularization (RNV) is a critical pathological feature of vision-threatening ocular diseases, such as diabetic retinopathy, retinopathy of prematurity, and wet age-related macular degeneration, presenting a persistent therapeutic conundrum. Current clinical treatments primarily rely on anti-vascular endothelial growth factor (VEGF) drugs and laser therapies, which face limitations including drug resistance, high costs, and potential damage to normal tissues. This underscores the need to develop novel therapeutic targets and cost-effective pharmacological interventions with improved safety profiles. Recent investigations highlight *Dendrobium* polysaccharides (DP), the primary bioactive components of the traditional medicinal herb *Dendrobium*, as promising multi-target therapeutic candidates. Studies have shown that *Dendrobium* polysaccharides significantly inhibits pathological angiogenesis by regulating the VEGF signaling pathway, inhibiting inflammatory response and oxidative stress, protecting the extracellular matrix, and reversing intestinal microecological disorders. This review systematically summarizes the structural and functional properties of DP, explores their mechanism of action and experimental evidence in retinal neovascularization, and analyzes their potential as a new therapeutic strategy for retinal diseases. This review also highlights the main limitations of current research: the uncertain relationship between the structure and activity of DP, the differences between pre-clinical models and human diseases, and the potential for structural optimization and the development of delivery systems.

## 1 Introduction

Retinal neovascularization (RNV) is an important factor in overall visual impairment, particularly among working-age adults and premature infants. This condition not only means advanced retinopathy, but is often associated with severe complications, including vascular leakage, hemorrhage, fibrotic marks and tractional retinal detachment, all of which profoundly compromise visual function (Arrigo and Bandello, 2021). RNV formation is an important pathological mechanism in chronic eye diseases such as age-related macular degeneration (AMD), retinopathy of prematurity (ROP), proliferative diabetic retinopathy (PDR), oxygen-induced retinopathy (OIR) and retinal vein occlusion (Grossniklaus et al., 2010). Dysregulation of the VEGF signaling pathway has been identified as a critical driver of neovascular pathogenesis (Penn et al., 2008). Current clinical interventions, including intravitreal anti-VEGF therapy and laser photocoagulation, effectively reduce vascular proliferation and promote neovascular regression. Despite these advances, significant challenges remain, such as the need for repeated intraocular injections, adverse systemic effects and suboptimal patient compliance. Moreover, resistance to anti-VEGF therapy observed in a subset of patients further exacerbates poor visual outcomes (Arrigo and Bandello, 2021). Therefore, identifying new therapies with higher efficacy and lower toxicity remains a priority in ophthalmic research.

Traditional Chinese medicine recognizes *Dendrobium* species as “eye strengthening and Yin strengthening” agents, their therapeutic applications being documented in the Materia Medica Compendium (Xie et al., 2023; Zhang et al., 2018). Several experimental studies have shown that natural compounds derived from *Dendrobium* have significant anti-angiogenic properties. Specifically, gigantol performs its therapeutic effects by inhibiting the AKT and ERK1/2 signaling pathways, effectively inhibiting cell migration and reproduction, as well as apoptosis and inflammatory responses of endothelial cells, ultimately inhibiting pathological angiogenesis in corneal and retinal tissues (Jiang et al., 2024; Liu B. et al., 2021). Benzyl compounds extracted from *Dendrobium* showed inhibitory effects on the ERK1/2-NF-κB signaling cascade in both *in vitro* and *in vivo* models. This substance ultimately protects the integrity of BRB in the DR by reducing inflammatory factors caused by microglial activation while upregulating the expression of tight junction proteins (claudine-1, occludin) and downregulating VEGF expression (Zhang et al., 2019). The aqueous extract of *Dendrobium* demonstrates attenuation of vascular endothelial inflammation through modulation of the TLR4/NF-κB/ICAM-1 axis (Chen T. E. et al., 2024). Eryanin, a bioactive component isolated from *Dendrobium*, showed significant anti-inflammatory and antiangiogenic effects in vitro and *in vivo* models by modulating the expression of components of the HIF-1α/VEGF signaling pathway (Yu et al., 2016). *Dendrobium* ethanolic extract also attenuated inflammation in the retina by inhibiting the NF-κB signaling pathway and restoring tight junction protein expression to physiological levels (Yu et al., 2015). Mechanistically, the extract exerts dual therapeutic effects by targeting VEGF/VEGFR2 signaling and inhibiting other pro-angiogenic factors (MMP-2/-9, PDGF-A/B, bFGF, IGF-1), thereby attenuating pathological angiogenesis in DR (Gong et al., 2014). Recent pharmacological advancements highlight the therapeutic potential of plant polysaccharides, particularly their multi-target efficacy and favorable safety profile, such as *Lycium barbarum* polysaccharides, tea polysaccharides, and *astragalus* polysaccharides (Chen L. et al., 2022). Among these, *Dendrobium* polysaccharides (DP), the main active ingredient of *Dendrobium* species, have been pharmacologically evaluated to exert antioxidant, anti-inflammatory and immunomodulatory bioactivities (Xie et al., 2023; Zhang et al., 2018). Emerging evidences further support the anti-angiogenic properties of DP. As a study found that different concentrations of DP improved oxidative stress and apoptosis in vascular endothelial cells induced by hyperglycemia, thereby preserving endothelial cell function (Chen et al., 2020). Further studies revealed that DP could inhibit the protein expression of angiogenic markers (CD31, CD34, and VWF) and downregulate pro-angiogenic proteins (VEGF, VEGFR2, and angiopoietin-1) (Yang, 2023). DP can inhibit pathological angiogenesis through multiple mechanisms, including modulating the VEGF signaling pathway, relieving inflammatory-oxidative cascades, protecting extracellular matrix integrity, and restoring gut microbial homeostasis. However, studies on the mechanism of action of DP in RNV are still relatively limited. This review summarizes the multi-targeted mechanism of action of DP in RNV formation, explores its potential as a novel therapeutic strategy for retinal diseases, and suggests strategic directions for future mechanistic studies through a systematic analysis of the existing literature.

## 2 Pathological mechanisms of retinal neovascularization

**FIGURE 1:**
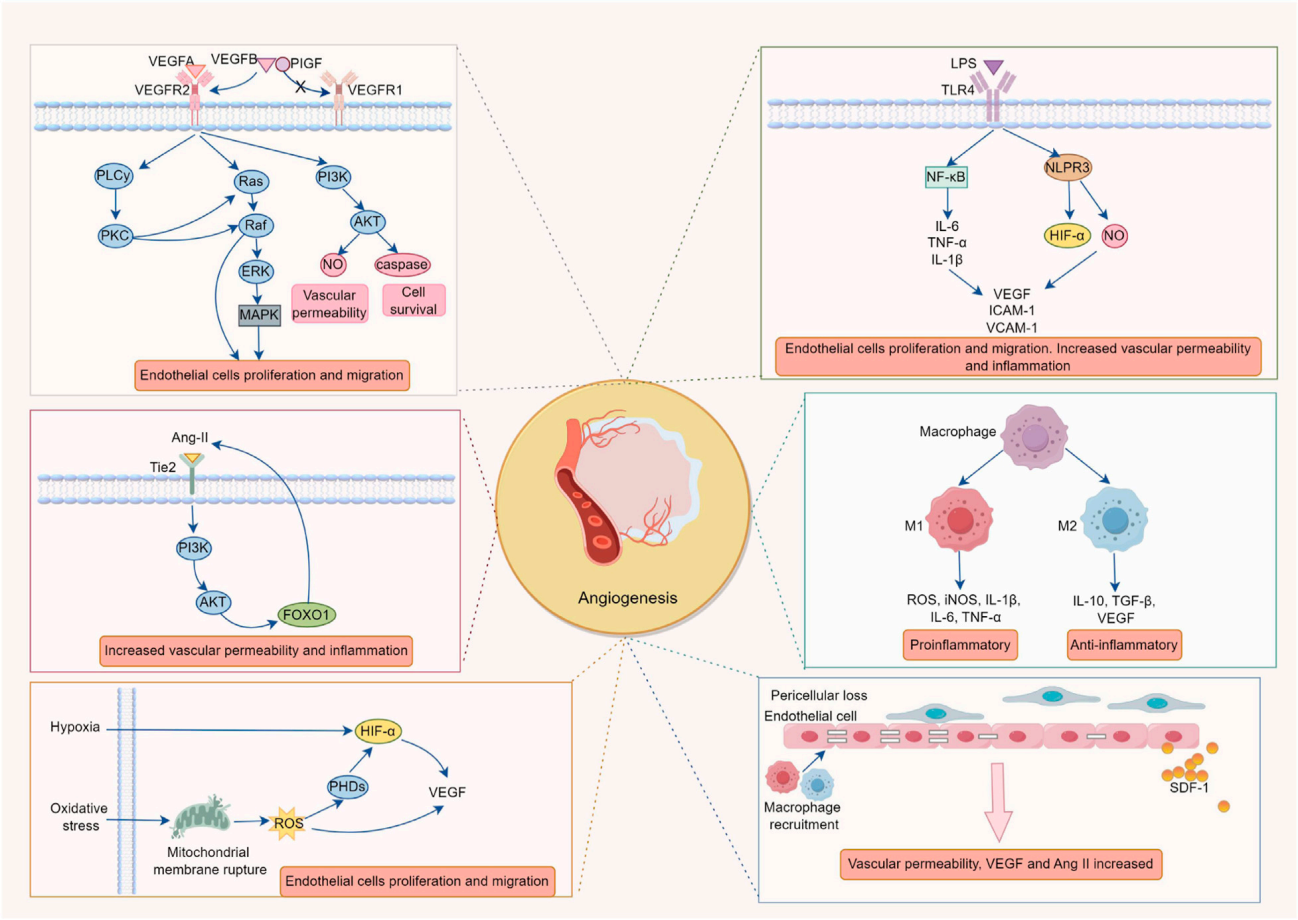
The Pathological Mechanisms of Retinal Neovascularization. The formation of retinal neovascularization (RNV) is driven by a multi-factorial cascade reaction, triggered by a vicious cycle of hypoxia, inflammation, and oxidative stress. The key mechanisms include: 1. The ischemic and hypoxic microenvironment activates the VEGF/VEGFR2 signaling axis *via* HIF-1α, in conjunction with the imbalance of AngII/Tie2 and disruption of the PDGF-B/PDGFRβ pathway, promoting endothelial cell proliferation, migration, and lumen formation while compromising the integrity of the blood-retinal barrier (BRB). 2. Chronic inflammation (mediated by NF-κB and the release of IL-6, TNF-α) and ROS bursts enhance HIF-1α stability and upregulate VEGF expression, further exacerbating endothelial cell activation and vascular leakage, thus intensifying the vicious cycle of worsening hypoxia → amplified inflammation → oxidative damage. 3. Polarization of M2 macrophages (secreting VEGF and IL-10) and abnormal recruitment of microglial cells release pro-angiogenic factors, which, in conjunction with the loss of pericytes, contribute to vascular instability and promote pathological angiogenesis. 4. Pericyte apoptosis, through the FOXO1-AngII positive feedback loop, increases endothelial cell sensitivity to VEGF, accelerating the formation of non-perfused areas and vascular leakage, ultimately leading to fibrosis and tractional retinal detachment.

RNV formation is a complex pathological process involving the interaction of multiple cell types, cytokines and signaling pathways, such as endothelial cell apoptosis, pericyte loss, immune cell dysfunction, activation of multiple growth factors and dysregulation of signaling pathways (Figure 1).

### 2.1 Regulatory network of angiogenesis

Angiogenesis is a complex biological process. Under normal physiological conditions, it remains relatively quiescent, but external stimuli can induce pathological angiogenesis to repair damaged tissues. Among these factors, VEGF plays a crucial role as the most potent pro-angiogenic factor. VEGF directly stimulates endothelial cell proliferation, migration, and lumen formation, significantly increasing vascular permeability (Fu et al., 2023). Its biological effects are primarily mediated through binding to membrane-bound receptors VEGFR1 and VEGFR2, with VEGFR2 being more abundantly expressed in endothelial cells. However, studies suggest that under physiological conditions, VEGF binding to soluble or membrane-bound VEGFR1 may inhibit VEGF-VEGFR2 signaling through higher affinity binding, exerting anti-angiogenic effects to maintain balanced regulation in the physiological state (Uemura et al., 2021; Wu et al., 2010). Under pathological conditions such as hypoxia and inflammation, the expression of the VEGF family is significantly upregulated, leading to VEGFR1 competitively binding with placental growth factor (PlGF) and VEGF-B, which in turn promotes the activation of VEGFR2 (Robciuc et al., 2016; Yang et al., 2013). The binding of VEGF to VEGFR2 continues to activate several downstream signaling pathways, including the PLCγ/PKC, Ras/Raf/ERK/MAPK, and PI3K/AKT signaling pathways. These pathways collectively regulate vascular permeability, promote endothelial cell proliferation and anti-apoptotic activity, and influence endothelial cell migration, actin reorganization, and cytoskeletal remodeling, ultimately leading to the formation of pathological neovascularization (Claesson-Welsh, 2012; Melincovici et al., 2018). The VEGF-VEGFR2 signaling cascade has been demonstrated to critically impair endothelial adhesion and intercellular connections, leading to increased vascular permeability and fluid extravasation, and subsequent breakdown of the blood-retinal barrier (BRB) (Simons et al., 2016). Although the VEGF-VEGFR2 signaling pathway is recognized as an important angiogenic pathway, the regulatory role of VEGFR1 is still controversial: most current evidence on the mechanisms of VEGFR1 inhibition is based on *in vitro* or animal models, and its regulatory role in human diseases remains unclear.

The fibroblast growth factor (FGF) serves as a pivotal regulator of vascular development and tissue regeneration. Mechanistically, FGF induces endothelial cell proliferation and migration while simultaneously facilitating pericyte recruitment, processes that collectively enhance vascular integrity through regulation of endothelial permeability and microvascular network formation (Seghezzi et al., 1998; Ucuzian et al., 2010). FGF1 and FGF2 isoforms have been shown to have significant proangiogenic effects on endothelial cells, they can not only promote angiogenesis independently, but also synergistically interact with VEGF through VEGFR2-FGFR1 complex formation to promote neovascularization (Hori et al., 2017; Melincovici et al., 2018). In addition, endothelial-derived platelet-derived growth factor B (PDGF-B) exerts angiogenic functions by binding to its receptor PDGFR, activating downstream signaling pathways similar to those induced by VEGF (Vimalraj, 2022), and participating in pericyte recruitment and new blood vessel formation (Tang et al., 2023). During tumor angiogenesis, abnormal PDGF signaling recruits pericytes to blood vessels, releasing pro-angiogenic factors that further stimulate endothelial cell migration, proliferation, and lumen formation, thereby accelerating lymphangiogenesis and invasion (Vimalraj, 2022). Angiopoietins (Ang-I and Ang-II) are also key factors in regulating angiogenesis and vascular remodeling. In endothelial cells, Ang-II binds to the Tie2 receptor, inhibits the PI3K/Akt pathway, and activates the forkhead box transcription factor FOXO1. FOXO1 activation further upregulates Ang-II, establishing a positive feedback loop that promotes angiogenesis, vascular permeability, and inflammation (Augustin et al., 2009; Kusuhara et al., 2018). In pathological conditions such as DR, AMD, and retinal vein occlusion, the expression of Ang-II is significantly upregulated, increasing endothelial cell sensitivity to pro-angiogenic, pro-permeability, and pro-inflammatory stimuli (Augustin et al., 2009; Regula et al., 2016; Watanabe et al., 2005). Under conditions of ischemia, hypoxia, and inflammation, not only are VEGF and HIF-1α highly expressed, but Ang-II also acts as an antagonist to Ang-I, disrupting Ang-I mediated vascular homeostasis, impairing endothelial and supporting cell interactions, and inducing endothelial cell apoptosis, vascular instability, and neovascular formation (Huang and Bao, 2004).

### 2.2 The malignant cycle of hypoxia-inflammation-oxidative stress

Under ischemic and hypoxic conditions, cells respond to environmental changes by upregulating HIF-1α, which activates a series of hypoxic response genes and induces HIF-1α-dependent VEGF transcription (Rattner et al., 2019). This results in increased capillary permeability, neovascular formation, and disruption of the BRB integrity. Clinical studies have demonstrated that intravitreal anti-VEGF therapy reduces circulating VEGF levels in patients and effectively decreases the release of inflammatory cytokines (Mastropasqua et al., 2018; Raczyńska et al., 2018), thus mechanistically linking chronic inflammatory cascades to retinal RNV pathogenesis. When exposed to external stimuli, various pattern recognition receptors (such as TLRs and NLRs) recognize exogenous or damaged molecules (e.g., LPS, CpG DNA, lipoproteins, flagellin) (Wang R. et al., 2023), activating intracellular inflammasomes and the NF-κB signaling pathway. This process triggers the release of numerous pro-inflammatory cytokines (e.g., IL-1β, IL-6, TNF-α) and other inflammatory mediators (e.g., HIF-1α, NO). While this response helps repair damaged tissue somewhat, it can also lead to sustained tissue damage, further activating endothelial cells. These cells secrete leukocyte adhesion molecules (e.g., ICAM-1, VCAM-1), cytokines, and chemokines, increasing VEGF expression (Fu et al., 2020; Heloterä and Kaarniranta, 2022), promoting endothelial cell proliferation and migration, and resulting in angiogenesis and neovascular formation in avascular regions.

Oxidative stress plays an important role in the development of RNV. When the antioxidant defense system is impaired or reactive oxygen species (ROS) are overproduced, an imbalance between ROS generation and clearance significantly increases intracellular oxidative stress levels. This is especially true when excessive activation of the mitochondrial electron transport chain results in ROS accumulation, directly damaging mitochondria and DNA and inducing endothelial cell apoptosis. ROS not only increases the transcriptional activity of HIF-1α, promoting VEGF expression, but also activates a series of pro-inflammatory responses, accelerating capillary permeability and neovascular formation (Cheung et al., 2010; Fu et al., 2020; Yang et al., 2022). Chronic oxidative stress also induces glial cell secretion of inflammatory factors, maintaining a persistent chronic inflammatory state in retinal cells, and ultimately promoting retinal neurodegeneration and microvascular lesions (Batliwala et al., 2017). Although the HIF-1α-VEGF axis is recognized as an important signaling pathway, most studies are based on acute hypoxia models and have not adequately modeled the dynamic process of hypoxia and repetitive reperfusion that occurs in chronic retinal disease. This may lead to an underestimation of the complexity of endothelial cell adaptation to hypoxia. The dual role of ROS in RNV, promoting angiogenesis and triggering cell apoptosis, is well established. Still, in current experimental systems, it is often not possible to separate the specific contribution of different forms of ROS (such as O_2_^−^, H_2_O_2_, H_2_O_2_, and OH^−^) at different pathological stages. This uncertainty at the molecular level limits the accuracy of targeted antioxidant therapy.

### 2.3 Mononuclear phagocyte system

Monocytes/macrophages, as key sources of angiogenic factors, play a significant role in RNV, particularly in PDR and OIR models (Tu et al., 2024). The chronic inflammatory microenvironment in retinal diseases is often accompanied by the infiltration of mononuclear phagocytes, including circulating monocytes/macrophages and resident microglia in tissues (Nathan and Ding, 2010; Rashid et al., 2019; Uemura et al., 2021). Studies have indicated that activated macrophages exhibit significant pro-angiogenic effects *in vivo* and *in vitro* (Hagbi-Levi et al., 2017). Upon stimulation, monocytes “polarize” into two macrophage subtypes: M1 and M2. M1 macrophages produce large amounts of ROS, inducible nitric oxide synthase (iNOS), IL-1β, IL-6, TNF-α, and other inflammatory mediators, exhibiting antimicrobial and pro-inflammatory actions while inhibiting angiogenesis. M2 macrophages, on the other hand, secrete anti-inflammatory mediators, growth factors, and pro-angiogenic factors (Nicholas and Mysore, 2021), such as IL-10, TGF-β, and VEGF, playing roles in anti-inflammation, tissue repair, and angiogenesis (Tu et al., 2024). In PDR and OIR models, increased expression of M2 macrophages is closely associated with the progression of pathological RNV, while the number of M1 macrophages is significantly reduced (Wang et al., 2024). Under hypoxic conditions, HIF-1α upregulates inflammatory cytokines (such as VEGF, IL-6, IL-8) and chemokines (such as MCP-1), promoting the recruitment and migration of peripheral microglia to the retina (Chen Z. Y. et al., 2024). This triggers the continued secretion of pro-inflammatory chemokines and cytokines, establishing a vicious cycle that promotes angiogenesis (Grossniklaus et al., 2002). It has been found that genetic ablation of the VEGFR1 TK structural domain in mice permitted normal vascular development but significantly inhibited VEGF-induced macrophage migration (Uemura et al., 2021), suggesting that activation of VEGFR1 also generates pro-inflammatory and pro-angiogenic mediators in macrophages and microglia (Carmeliet et al., 2001; Crespo-Garcia et al., 2017).

### 2.4 Loss of pericytes

Pericytes are mesenchymal cells that surround the endothelial cells of capillaries and work together with endothelial cells to maintain vascular integrity and stability. A decrease in the ratio of pericytes to endothelial cells leads to increased vascular permeability and cell-free capillary formation, which exacerbates microangiomas, vascular occlusion, and local retinal hypoxia, further promoting RNV formation (Mrugacz et al., 2021). Pericyte loss is usually induced by advanced glycation end products (AGEs), oxidative stress, inflammation and other factors, which destroy vascular structure through an apoptosis mechanism and play a key role in the development of blinding eye diseases (Han et al., 2023). In patients with DR, pericyte loss compromises endothelial cell integrity, and internal BRB function is consequently disrupted, inducing retinal microangiopathy, including microaneurysms, oedema, haemorrhage, decellularised capillaries and areas of capillary non-perfusion (Arboleda-Velasquez et al., 2015; Kusuhara et al., 2018), and this idea has also been supported in animal models of diabetes mellitus (Valdez et al., 2014). Relevant studies have shown that pericyte loss not only disrupts the tight junctions between endothelial cells, but also increases the sensitivity of endothelial cells to VEGF, thus leading to a positive feedback loop between FOXO1 and Ang II (Park et al., 2017), which further promotes abnormal vascular proliferation, vascular leakage, and inflammatory responses. Vascular leakage and occlusion in turn exacerbate retinal ischaemia, upregulate HIF-1α and further promote the expression of VEGF and Ang-2 (Rattner et al., 2019). Pericyte loss also releases chemokines such as stromal cell-derived factor-1 (SDF-1), which induces inflammatory responses in endothelial cells, macrophages and microglia through perivascular infiltration. VEGF is secreted to activate VEGFR2 in endothelial cells and a series of downstream signaling cascades, further damaging vascular integrity (Ogura et al., 2017). In addition, weak vascular walls and abnormal blood flow shear forces in the bloodstream caused by pericyte loss further aggravate local vasodilation (Tian et al., 2022). Due to the lack of pericyte coverage, neovascularization can be prone to hemorrhage and fibrosis, eventually leading to a tractional retinal detachment (Zhang J. et al., 2025).

## 3 Characteristics and biological activities of *Dendrobium* polysaccharides

*Dendrobium* spp., which has a long medical history in traditional Chinese medicine, includes various varieties such as *Dendrobium nobile Lindl*, Dendrobium officinale Kimura & Migo, and Dendrobium huoshanense Z.Z.Tang & S.J.Cheng as documented in the Chinese Pharmacopoeia (2020 edition) (Zhang et al., 2018). These botanicals are rich in bioactive components, mainly polysaccharides, flavonoids, alkaloids, phenols and amino acids, whose pharmacological properties and health-beneficial functions have been extensively studied. It is important to note that the main polysaccharides are composed of carbohydrates called glycans, which are complex macromolecules composed of more than 10 monosaccharide units linked together. These glycans have various biological activities, including lowering blood sugar levels, immunomodulatory properties, antioxidants and gastrointestinal protection (Fan et al., 2023; Gong et al., 2014; Wang H. Y. et al., 2019). DP is mainly obtained by extraction with water, alcohol, ultrasound, *etc.* Their structure is complex and variable, and they are usually found in pyranose form, consisting mainly of glucose (Glcp), mannose (Manp), galactose (Galp), arabinose (Arap), xylose (Xylp), rhamnose, and other monosaccharides. The content of polysaccharides varies from genus to genus depending on factors such as climate, temperature, humidity and soil. The structure of polysaccharides varies from genus to genus, and the differences lie mainly in the type and proportion of monosaccharides, the molecular weight, and the type of glycosidic bonds and their linkages, which can lead to different pharmacological activities (Bu et al., 2019). Different extraction methods can also affect the content and pharmacological effects of different polysaccharides by changing the proportion of simple sugars rather than their type. For example, ultrasonic extraction leads to a decrease in the molecular weight of polysaccharides and the degradation of their main chains, a decrease in viscosity, and an increase in water solubility and biological activity (He et al., 2018). Most current studies of polysaccharides from Dendrobium officinale Kimura & Migo (DOP) and Dendrobium huoshanense Z.Z.Tang & S.J.Cheng (DHP) were identified as glucomannan bases containing 1,4-β-D-Manp and 1,4-β-D-Glcp units. The molecular mass of DOP ranged from 0.46 KDa to 2.27 × 10^6^ kDa and that of DHP from 2.55 KDa to 770 KDa, with different degrees of acetylation or branching of the glucan residues (Hsieh et al., 2008; Deng et al., 2016; Chen W. et al., 2021; Li et al., 2022; Wu et al., 2023; Zhang M. et al., 2024). Most of the *D. nobile Lindl* (DNP) polysaccharides have been identified as glucomannan with glycosidic bonds such as α-(1→4), α-(1→6), β-(1→4), β-(1→6) and others, including the acetylation of mannose residues at various sites, with or without branched chains (Luo et al., 2009; Chen H. et al., 2022; Li et al., 2024). The keyword “*Dendrobium* polysaccharides” was searched in PubMed, Web of Science, Google Scholar, CNKI and Wanfang databases to retrieve experimental articles on the assessment of the effectiveness of DP in the past 10 years, and summarized and analyzed in Table 1.

**TABLE 1:** Types of *Dendrobium* polysaccharides and their related functions.

*Dendrobium* genus	Polysaccharide name	Extraction site	Main component monosaccharides	Molecular weight	Structural features	Function	References
*Dendrobium nobile* Lindl	DNP1, DNP2	*Dendrobium* powder	DNP1: mannose (75.86%), glucose (24.14%); DNP2: mannose (72.32%), glucose (27.68%)	67.72 KDa; 37.45 KDa	DNP1: β-1,4-D-Manp, β-1,4-D-Glcp, Acetyl: C-2 or C-3 sites of mannose residues	Decreased NO and pro-inflammatory factor secretion and promoted anti-inflammatory factor (IL-10) secretion	Chen et al. (2022)
	DNP	stems	Glucose, galactose, mannose, arabinose, xylose, rhamnose (molar ratio 117.96:31.76:30.76:2.80:2.20:1.00)	87.6 KDa	Main chain: (1 → 6)-α-D-Glcp, (1 → 6)-α-D-Galp, branches: (1 → 4)-α-D-Glcp, (1 → 4)-α-D-Manp	Hypoglycemia, up-regulation of SIRT1 attenuates oxidative stress and inhibits apoptosis.	Lei et al. (2022)
	JCS1	stems	Glucose, mannose, xylose, arabinose (molar ratio 40.2:2.3:1.7:1.0)	23 KDa	Main chain: (1→4)-β-D-Manp, (1→4)-α-D-Glcp, branch: (1→4)-α-D-Glcp at C6	Neuroprotective effect	Jin et al. (2017)
	JCS1S2	stems	Glucose, mannose, xylose, arabinose (molar ratio 40.2:2.3:1.7:1.0)	56.2 KDa	Main chain: 1, 4-β-D-Manp, 1, 4-α-D-Glcp, branch: sulfation of β-Manp and α-Glcp residues at C-6 site	Blocking VEGF signaling, anti-angiogenic effect	Wang Z. et al. (2019)
	DNPE6	*Dendrobium* powder	Glucose, mannose, galactose (molar ratio 11:3:3)	3.01 KDa	→1)-Manp-(3, 6→, T-Galp, and →1)-Glcp-(4→	Antiviral effects	Li M. Z. et al. (2020)
*Dendrobium officinale* Kimura & Migo	DOP-W1, DOP-W2	stems	DOP-W1: mannose, glucose (molar ratio 10.75:1.00); DOP-W2: mannose, glucose (molar ratio 8.82:1)	389.98 KDa; 374.11 KDa	Main chain: (1 → 4)-β-D-Manp, (1 → 4)-β-D-Glcp, branches: terminal Manp of O-3 and O-6 Composition	Promote macrophage proliferation, phagocytosis and immunomodulatory effects	Tao et al. (2019)
	DOP	stems	Mannose, glucose, arabinose (molar ratio 40.2:8.4:1)	130 KDa	Main chain: (1 → 4)-β-D-manp, (1 → 4)-β-D-Glcp, Branches: (1 → 3)-Manp, (1 → 3)-Glcp, Acetyl: (1 → 4)-linked Manp and Glcp at O-2	Enhance immunity and lower blood sugar	Hua et al. (2004)
	DOPS-1	stems	Mannose, glucose, galacturonic acid (molar ratio 3.2:1.3:1)	1,530 kDa	(1→4)-β-D-Glcp, (1→4)-β-D-Manp and 2-O-Acetyl-(1→4)-β-D-Manp	Antioxidant, tumor cell inactivation	Wang et al. (2021)
	DOP	stems	Mannose, glucose, arabinose (molar ratio 5.55:1:0.12)	190 kDa	Main chain: (1 → 4)-β-D-manp, (1 → 4)-β-D-Glcp, Branches: (1 → 3)-Manp, (1 → 3)-Glcp, Acetyl: (1 → 4)-linked Manp and Glcp at O-2	Regulation of intestinal flora, improvement of intestinal barrier function, immunomodulation, cancer intervention, regulation of metabolism, anti-inflammation	Liang et al. (2019), Liu et al. (2020)
	DOP	stems	Mannose, glucose (6.9:1)	312 kDa	Main chain: (1 → 4)-β-D-manp, (1 → 4)-β-D-Glcp, Acetyl: O-2 or O-3 sites of mannose residues	Reduce blood glucose, blood fat, improve insulin resistance and bile acid, amino acid metabolism disorders, antioxidant, hepatoprotective effects	Yang K. et al. (2020)
	LDOP-1	leaf	Mannose, galacturonic acid, glucose, galactose, arabinose (molar ratio 2.0:1.7:1.3:1.6:0.7)	91.8 KDa	1,6-α-D-Glup, 1,4- α-D-Manp	Inhibits TLR4/NF-κB signaling pathway, anti-inflammatory, down-regulates Bax/Bcl2 ratio, inhibits caspase 3 activation, anti-apoptosis	Yang J. et al. (2020)
	DOP	leaf	Mannose, glucose (4.211:1)	510 KDa	-	Anti-osteoporosis, inhibits Nrf2/Keap1 interaction, inhibits Nrf2 ubiquitination, promotes Nrf2 nuclear translocation	Wang Y. et al. (2023)
	DOP	stems	Mannose, glucose (molar ratio 1.38:1.00)	395 KDa	D-Glcp、D-Manp	Inhibition of JNK protein expression, up-regulation of PI3K and Glut2 expression downstream of IRS1, antidiabetic and amelioration of insulin resistance	Wang C. et al. (2018)
	DOP		Glucose, mannose (molar ratio 3.52:1.00)	1,249.382 kDa	(1 → 4)-α-D-Manp	Antioxidant, inhibits glial cell activation, inhibits NF-κB signaling pathway and neuroinflammation, improves gut microbiota disorders	Lei et al. (2023)
	S32S	stems	Xylose, arabinose, glucose (molar ratio 73.6:10.3:7.4)	54 KDa	Main chain: (1 → 4)-β-D-Manp, (1 → 4)-α-D-Glcp, branch: (1 → 4)-α-D-Glcp residues at C-6, β-Manp and α-Glcp residues sulfated at C-6	Anti-angiogenic, inhibits endothelial cell migration	Yue et al. (2017)
*Dendrobium huoshanense* Z.Z.Tang & S.J.Cheng	DHP	stems	Mannose, glucose, galactose, arabinose (molar ratio 225.57:136.40:3.17:2.03)	27.394 KDa	-	Regulates intestinal flora imbalance and metabolite levels, hepatoprotective	Ma et al. (2023)
	DJP	stems	Mannose, glucose (molar ratio 2.88:1.00)	159 KDa	Main chain: β-(1 → 4)-D-Glcp, β-(1 → 4)-D-Manp, Acetyl: 3-O-acetyl-β-(1 → 4)-D-Manp	Inhibition of PI3K/AKT, NF-κB pathway and activation of Nrf2 pathway, anti-inflammatory, antioxidant, restoration of Th17/Treg balance	Ye et al. (2024)
	DHP-2W	stems	Mannose, glucose (molar ratio 75.81:24.19)	508.934 kDa	Main chain: (1 → 4)-β-D-Manp, (1 → 4)-β-D-Glcp	Anti-inflammatory, antioxidant, inhibits apoptosis, activates AMPK, balances energy metabolism	Zhang X. et al. (2024)
	GXG, dGXG	stems	GXG: glucose, xylose, galactose (molar ratio 2.85:2.13:1.00); dGXG: glucose, xylose, galactose (molar ratio 2.01:1.50:1.00)	1,780 KDa	GXG:1,4-Glcp, 1,6-Glcp, 1,4,6-Glcp, 1,4-Galp, 1,3,6-Galp, 1-Galp, 1,4-Xylp, 1,2,4-Xylp,1-Xylp:dGXG:1,4-Glcp, 1,6-Glcp, 1,4,6-Glcp, 1,3,6-Galp, 1-Galp, 1,4-Xylp, 1,2,4-Xylp,1-Xylp	Regulates intestinal flora and metabolites, protects the intestinal barrier, regulates the development of immune cells and enhances the immune response	Xie et al. (2019)
*Dendrobium wardianum* R.Warner	DWPP-I	stems	Mannose (76.66%), Glucose (22.85%)	96.8 KDa	Main chain: (1 → 4)-β-D-Glcp, (1 → 4)-β-D-Manp, acetyl: O-2 or O-3 position of (1 → 4)-β-D-Manp	Inhibit tumor cell growth and proliferation	Ye et al. (2021)
*Dendrobium chrysotoxum* Lindl	DCP	stems	Galactose, glucose, mannose (molar ratio 0.04:1.00:0.42)	-	-	Antioxidant	Pan et al. (2014)
*Dendrobium devonianum* Paxton	DVP-1	stems	Mannose, glucose (molar ratio 10.11:1.00)	95.2 KDa	(1 → 4)-β-D-Glcp, (1 → 4)-β-D-Manp, Acetyl: O-2 or O-3 or O-6 position of (1 → 4)-β-D-Manp	Stimulation of macrophage activation through TLR4 and promotion of macrophage proliferation	Wu et al. (2019)
	DDP	*Dendrobium* powder	Mannose, glucose (molar ratio 29.61:1.00)	399 KDa	Main chain: 1,4-β-D-Manp, 1,4-β-D-Glcp, Acetyl: O-2 or O-3 sites on mannose residues	Promote macrophage function	Deng et al. (2018)
*Dendrobium flexicaule* Z.H.Tsi, S.C.Sun and L.G.Xu	DFP	stems	Mannose (79.89%), glucose (19.05%), xylose (0.42%)	367.478 KDa	-	Protects the gastric barrier, an antioxidant, and an anti-inflammatory	Wang et al. (2025)
*Dendrobium hancockii* Rolfe	PDH-1, PDH -2, PDH-3	stems	PDH-1: galactose, mannose (molar ratio 11.5:1); PDH-2: galactose, mannose (molar ratio 10.1:1); PDH-3: galactose, mannose (molar ratio 49:1).	40.4 KDa; 8.35 Da; 8,780 KDa	Main chain: 4-β-D-Manp, (1 → 4)-β-D-Manp, O-2 and/or O-3 positions substituted with O-Ac groups	Antioxidant, antimicrobial	Wang et al. (2022)

## 4 Mechanism of *Dendrobium* polysaccharides in inhibiting retinal neovascularization

### 4.1 Inhibition of the VEGF signaling pathway

Under retinal ischemia and hypoxia, the abnormal activation of the HIF-1α/VEGF signaling axis is one of the core mechanisms that promote the formation of pathological RNV. In an ischemic hypoxia model, DP was found to downregulate HIF-1α expression in a dose-dependent manner (Li and Hong, 2020), which might involve the classical PHD2/HIF-1α pathway, whereby the activation of prolyl hydroxylase 2 (PHD2) accelerates oxygen-dependent HIF-1α proteasomal degradation (Majmundar et al., 2010). Under hypoxic conditions, as the stability and accumulation of HIF-1α decrease, the binding of HIF-1α to the hypoxia-responsive element (HRE) in the promoter region of VEGF is also attenuated, which reduces transcriptional activation of VEGF and inhibits angiogenesis. Most current studies on DP have indirectly exerted anti-angiogenic effects based on anti-inflammatory and antioxidant properties. Researchers isolated the JCS1 polysaccharide from the stem of *D. nobile Lindl* and found that its backbone consists of (1→4)-β-D-Manp and (1→4)-α-D-Glcp (Wang Z. et al., 2019). They then modified the monosaccharide residues to C-6 in the main chain by sulfation to obtain a new derivative, JCS1S2. JCS1S2 showed significant inhibitory effects in wound healing and angiogenesis assays *in vitro* by antagonizing the low affinity of VEGFR2 and binding directly to VEGF, thus blocking VEGF/VEGFR2 signaling. At the same time, this polysaccharide reduces the phosphorylation of VEGFR2 and ERK and decreases the expression of VEGF and its transcription factor AP-1, thereby inhibiting neovascularization. Notably, JCS1S2 inhibited the migration of endothelial cells without affecting their proliferation (Bai et al., 2025). injected JCS1S2 into the vitreous of rats with OIR and found that the expression of the VEGF family, especially VEGF-A, VEGF-B, VEGF-D, VEGFR1, and VEGFR2, was significantly reduced, and neovascularization in the retina and areas not subject to perfusion was also significantly reduced after treatment. Other researchers have studied sulfated polysaccharides and angiogenesis (Yue et al., 2017). They extracted the S32 polysaccharides from the stem of Dendrobium officinale Kimura & Migo and analyzed their structures, which consist mainly of 1,4-β-D-xylp and 1,2,4-β-D-xylp, and modified C-2 or C-3 of the 1,2,4-β-D-xylp residues by sulfation to obtain the sulfated S32S polysaccharides. In this study, S32S showed a dose-dependent inhibitory effect on tube formation by HMEC-1 cells and significantly inhibited endothelial cell migration at a low concentration of 0.29 μM. Although S32S has potent antiangiogenic effects, it does not have significant cytotoxicity. This class of polysaccharides has a great influence in fighting against angiogenesis, is potentially valuable, and deserves more attention.

### 4.2 Anti-inflammatory and immune microenvironment remodelling

DP has significant anti-inflammatory efficacy, which reduces the infiltration of inflammatory cells such as neutrophils and macrophages by inhibiting the activation of inflammatory signaling pathways such as NF-κB and decreases the release of pro-inflammatory factors such as TNF-α and IL-6 (Li, 2016; Li et al., 2016) as well as promotes the expression of anti-inflammatory factors such as IL-10 (Xie et al., 2022), which inhibits the activation of the transcripts of pro-angiogenesis-related genes that are associated with inflammation. DP also reduces retinal inflammatory microenvironment by inhibiting NLRP3 inflammasome activation, reducing the inclusion and activation of caspase-1/GSDMD, and reducing the release of pro-inflammatory cytokines (e.g., IL-1β) to reduce macrophage damage caused by the pathogen (Zhang et al., 2022). This process not only alleviates cell damage due to the inflammatory response but also enhances the activity of immune cells and downregulates VEGF expression, thereby reducing its stimulating effect on angiogenesis. In addition to inhibiting inflammatory responses, DP has been shown to play an important role in regulating immune cell function. Studies have shown that DP affects microglia polarization, promotes conversion from pro-inflammatory to anti-inflammatory phenotypes, reduces the secretion of pro-inflammatory cytokines, improves immune regulation, and increases vascular permeability and apoptosis (Feng et al., 2019). DP may also have direct immunomodulatory effects by binding to polysaccharide receptors on the surface of immune system cells (e.g., macrophages or lymphocytes), such as the mannitol receptor (Tao et al., 2019; Wu et al., 2019), enhancing lymphocyte proliferation and promoting macrophage activation and phagocytosis (Bai et al., 2025). DP can also promote the secretion of interferon-γ (IFN-γ), IL-12, IL-10 and other factors by immune cells to inhibit angiogenesis (Meng et al., 2013; Xie et al., 2022). Within adaptive immunity frameworks, DP can selectively regulate the secretion of Th1-type cytokines, elevate CD4+/CD8+ T lymphocytes ratios, and stimulate non-specific immunoglobulin synthesis (IgA, IgM, IgG), collectively improving systemic immune responsiveness (Li Z. et al., 2020; Wang K. et al., 2018). In particular, DP increases the immune activity of monocytes by activating the ERK and NF-κB signaling pathway, which manifests itself by stimulating immune cell proliferation, increasing cytokine secretion, NO and phagocytosis synthesis, and ultimately immune competence at the tissue level (Wei et al., 2016).

### 4.3 Antioxidant effects and mitochondrial protection

As a powerful natural antioxidant, DP demonstrates potent antioxidant properties through mechanisms related to scavenging free radicals and maintaining calcium homeostasis. They can effectively mitigate damage from intracellular oxidation by suppressing lipid peroxidation processes, evidenced by reduced malondialdehyde (MDA) accumulation. Concurrently, they increase cellular antioxidant protection through activating the superoxide dismutase (SOD) and glutathione (GSH) systems (Fan et al., 2020; Guo et al., 2024; Li B. et al., 2021). This mechanism preserves endothelial integrity by inhibiting apoptotic pathways and relieving inflammatory cascades caused by oxidative stress. Subsequent studies indicated that the antioxidative effect of DP may be closely related to the activation of the Nuclear factor E2-related factor 2 (Nrf2) pathway and downstream antioxidant enzymes, which can reduce the damage caused by ROS (Xu et al., 2022). Nrf2 is a key transcription factor involved in the regulation of the cellular antioxidant response. DP activates the Nrf2-Keap1 signaling pathway (by increasing Nrf2 expression in the nucleus and decreasing Keap1 expression in the cytoplasm) and triggers dissociation of the Nrf2-Keap1 complex, thereby enabling Nrf2 to avoid Keap1-mediated ubiquitination degradation (Lin et al., 2018). After the activation of Nrf2 nuclear translocation, it binds to the antioxidant response elements (ARE) and encodes transcriptional expression of antioxidant enzyme genes, thereby regulating intracellular antioxidant synthesis and directly inactivating ROS to reduce oxidative damage (Xu et al., 2022; Zhao et al., 2019). Under hyperglycemic conditions, inducible nitric oxide synthase (iNOS) triggers excessive NO production, which rapidly reacts with superoxide anion (O^2−^) to generate peroxynitrite (ONOO^−^), a cytotoxic oxidant that has a more potent oxidative effect than conventional reactive oxygen species. Experimental evidence suggests that DP attenuates oxidative cascades by down-regulating iNOS expression and inhibiting AGE formation (Fan et al., 2020).

**FIGURE 2:**
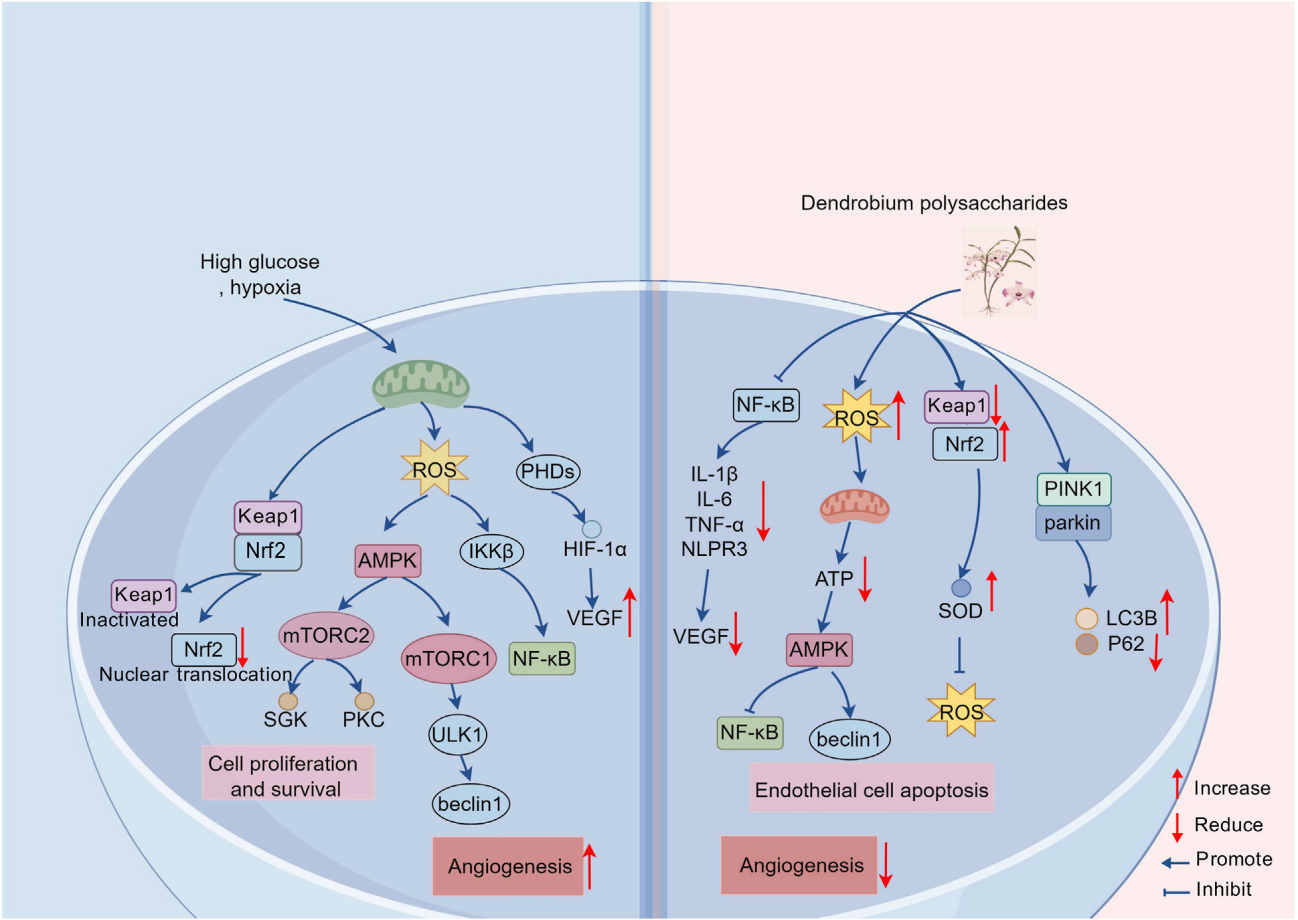
Schematic representation of anti-inflammatory, antioxidant and immune-enhancing properties of DP. DP demonstrates multimodal therapeutic effects through coordinated molecular interventions. They attenuate inflammatory cascades and oxidative stress while suppressing VEGF expression, thereby inhibiting vascular endothelial cell proliferation and migration. Enhanced phagocytic capacity of immunocytes is concurrently observed, coupled with angiogenesis suppression *via* mechanisms of macrophage protection and inflammatory pathway inhibition. It stimulates the expression of Nrf2 and reduces the activity of Keap1, activating the Nrf2-Keap1 pathway and prompting Nrf2 to escape from the ubiquitination degradation mediated by Keap1, thus binding to the downstream elements of the antioxidant response after nuclear translocation and stimulating the production of antioxidant enzymes sufficient for the elimination of ROS. The polysaccharides promote cellular homeostasis through *PINK1/Parkin*-mediated mitophagy activation, effectively counteracting ROS-induced damage. Paradoxically, controlled ROS elevation induces metabolic reprogramming *via* AMPK pathway activation, which suppresses ATP biosynthesis while potentiating autophagic flux. This dual regulatory mechanism culminates in endothelial cell apoptosis through excessive autophagy induction.

Mitochondria as the core regulators of energy metabolism in cells, and their dysfunction is often associated with oxidative stress and cellular damage. DP has been found to significantly increase the expression of mitochondrial autophagy-related proteins such as PINK1, Parkin, and LC3B, reducing the accumulation of the P62 autophagy receptor (Li H. et al., 2023). This not only scavenges ROS to resist oxidative damage, but also maintains intracellular metabolic homeostasis by promoting mitochondrial autophagy to eliminate functionally impaired mitochondria, which improves insulin resistance and repairs DNA damage. Notably, the protective effects of DP on mitochondrial function were also involved in increasing the activity of the mitochondrial respiratory chain complex, improving the AMPK signaling pathway and improving the efficiency of the tricarboxylic acid cycle, which ultimately inhibited neuronal apoptosis effectively (Chen L. et al., 2023). However, the mechanism of action of DP is significantly heterogeneous in some specific pathological situations. In the study of colon cancer CT26 cells, DP were found to be able to interfere with mitochondrial function by stimulating ROS bursts, lowering mitochondrial membrane potential and inhibiting ATP synthesis, while activating the AMPK-mTORC1-ULK1 pathway to cause excessive autophagy in cells, inhibit tumor cell proliferation and promote their death (Zhang et al., 2021) (Figure 2).

### 4.4 Regulation of extracellular matrix remodeling

The remodeling of extracellular matrix (ECM) and the destruction of dynamic balance play a central role in the formation of RNV. The composition of ECM includes components such as fibronectin, collagen, and laminin, and these molecules not only provide structural support for tissues but also regulate the migration, proliferation, and invasion of endothelial cells. However, pathological conditions, especially metabolic abnormalities such as hyperglycemia, can induce abnormal deposition of ECM components, leading to capillary basement membrane thickening (Yang and Liu, 2022). The thickened basement membrane enhances the sensing of mechanical tension of endothelial cells and promotes their proliferation and migration by enhancing the activity of integrin-focal adhesion kinase (FAK) signaling pathway (Katoh, 2024), thus providing favorable conditions for pathological RNV. In a therapeutic study targeting renal fibrosis (Shi et al., 2023), *Dendrobium* polysaccharide showed a significant reduction in ECM components, such as collagen I, collagen IV, and fibronectin, thereby alleviating the over-deposition of abnormal ECM and improving basement membrane thickening in a dose-dependent manner. The study also found that DP inhibited the expression of transforming growth factor beta 1 (TGF-β1) and connective tissue growth factor (CTGF), thereby reducing collagen synthesis. This suggests that DP can help to maintain the dynamic balance between ECM synthesis and degradation.

The excessive deposition of ECM and basement membrane thickening promotes increased expression and activity of matrix metalloproteinases (MMPs) during angiogenic processes. MMPs mediate the proteolytic almost all components of the ECM, thereby destabilizing the basement membrane’s structural organization and establishing permissive microenvironments for endothelial migration and subsequent angiogenic progression (Cabral-Pacheco et al., 2020). In endothelial cells, oxidative stress can promote the expression and activation of MMPs, promoting the degradation of ECM components. In inflammatory vascular lesions, the loss of tissue MMP inhibitors (TIMPs) is considered an important factor in vascular lesions. The imbalance between MMPs and TIMPs affects the degradation and innovation of ECM components and is considered a potential biomarker for diabetes and its complexity (Cabral-Pacheco et al., 2020; Fiorentino et al., 2013). Mediation of DP has been shown to significantly reduce the expression levels of MMP-1 and MMP-3, reduce collagen degradation and reduce the pro-degradation effect of MMPs by eliminating ROS, thus maintaining ECM stability and significantly inhibiting excessive endothelial cell migration and angiogenesis (Guo et al., 2024). In an ethanol-induced gastric ulcer study, SOD activity increased and MDA content decreased in gastric mucosal tissues treated with DP, while the levels of MMP-2, MMP-9, p-JNK, and p-ERK proteins were also significantly descreased. This also verified the effect of DP on the regulation of MMPS expression (Zhang et al., 2018). In another experiment with human skin fibroblasts in a hyperglycemic environment, DP increased collagen and TIMP2 levels and decreased MMP2 expression in a concentration-dependent manner (Liu F. Y. et al., 2021). This suggests the role of DP in the regulation of MMP2 and TIMP expression. In addition, membrane metalloproteinase type 1 (MT1-MMP) has also received special attention in angiogenesis (Sounni et al., 2002). This study demonstrates that MT1-MMP promotes endothelial cell migration through proteolytic degradation of the ECM and stimulates angiogenesis through VEGF expression modulation, providing a new perspective on molecular targets of DP for RNV inhibition. While there are still few studies on the regulation of TIMPs by DP, available research shows that the expression of TIMPs is regulated by oxidative stress and inflammatory responses (Amălinei et al., 2010). Unfortunately, the regulation of MMP and TIMP by DP has rarely been discussed in the field of ophthalmology, but the above studies indicate that DP may enhance the dynamic balance between MMPs and TIMPs and promote the regulation of ECM homeostasis, which may shed light on the mechanism of angiogenesis inhibition.

### 4.5 Intervention of retinal vascular diseases through the “gut-eye” axis

Systematic links between the gut microbiota and eye diseases are gradually being discovered (Thakur et al., 2022; Tîrziu et al., 2024). The intestinal flora and its metabolites can break through the intestinal mucosal barrier and enter the circulation through the cascade of the “intestinal barrier-circulation-target organ”, causing endotoxicity and systemic metabolic imbalance, and ultimately leading to pathological damage of the distal organs (e.g., the retina). In pathological states, when the balance of probiotic/pathogenic bacteria in the intestine is disturbed, not only does the increase in intestinal permeability cause localized inflammation, but the microorganisms can also cause extravagant inflammation throughout the body and reside in the intraocular lumen, which can lead to a variety of oxidative damage, Inflammation, immunodeficiency, metabolic disorders and other ocular events (Bai et al., 2023; Deng et al., 2021). In numerous human and animal studies, GM-derived metabolites, such as lipopolysaccharide (LPS), short-chain fatty acids (SCFAs), bile acids (BAs), and branched-chain amino acids (BCAAs), are involved in the regulation of retinal immune responses, energy metabolism homeostasis, and cellular autophagy through multiple targets, thereby affecting retinal angiogenesis (Xue et al., 2021). LPS is a characteristic metabolite of Gram-negative bacteria and is produced in increased amounts in a variety of ocular diseases, mainly due to increased abundance of *Bacteroides, Proteobacteria, Enterobacteria* and *Escherichia* (Qin and Zou, 2022). LPS exhibits dual pathological effects during retinal angiogenesis: on the one hand, as a pattern recognition receptor agonist, it specifically activates TLR4 on the surface of the retinal microvascular system and glial cells, triggering a cascade amplification of the NF-κB signaling pathway, which promotes downstream inflammatory factor expression (Xu et al., 2016). On the other hand, it significantly upregulates VEGF transcriptional expression through an inflammatory mechanism, induces pathologic angiogenesis, and disrupts BRB integrity, accelerating retinal microcirculatory dysfunction (Wang et al., 2020). BCAAs, including leucine, isoleucine, and valine, have high circulating levels due to increased GM synthesis in disease states and decreased metabolism. BCAAs can gradually dissolve retinal neurovascular device structures by activating the mTORC1 signaling pathway, exacerbating insulin resistance and oxidative stress, and inhibiting mitochondrial autophagy and promoting apoptosis in neurons and endothelial cells (Gong et al., 2022). GM produces SCFAs through the digestion of dietary vitamins, mainly acetate, propionate and butyrate, which have a significant role in defense against pathogenic bacteria and the development of the host immune system (Xue et al., 2021). It promotes intestinal GLP-1 secretion through activation of G protein-coupled receptors (GPR41/43) and inhibits histone deacetylase (HDAC) activity, promotes the differentiation of regulatory T cells (Treg), which modulates the body’s inflammatory response and glucose metabolism, and inhibits Th17 cell-mediated autoimmune retinal damage (R Muralitharan et al., 2025; Zhang Y. et al., 2025). Intravitreal injection of sodium butyrate significantly reduced the size of choroidal neovascularization (CNV) lesions in a mouse model of laser-induced CNV, and sodium butyrate nanoparticles also showed antiangiogenic activity in a chicken chorioallantoic membrane (CAM) experiment (Ciurariu et al., 2025). Concurrently, in a high glucose experiment with human retinal endothelial microvascular cells (HRMEC), sodium butyrate was also found to inhibit the ROS/NF-κB/NLRP3 signaling pathway and angiogenesis (Cao et al., 2025). Bile acids (BAs), whose conversion from primary (PBAs) to secondary (SBAs) is accomplished by the GM in the gut, have an important role in the absorption of dietary lipids and fat-soluble vitamins, and be neuroprotective and antiangiogenic in a variety of retinal diseases, such as DR and ROP (Win et al., 2021). SBAs reduce mitochondrial membrane potential and inflammatory responses by activating the G-protein-coupled receptor (TGR5) and the nuclear receptor (FXR), reducing Ca^2+^ overload and ROS bursts, inhibiting proinflammatory gene expression by antagonizing NF-κB and AP-1, and negatively regulating NLRP3 production, thereby reducing retinal Müller cell apoptosis, retinal vascular leakage, and inflammatory responses (Bertolini et al., 2022; Li J. et al., 2023). *In vitro* studies, lithocholic acid (LCA), glycocholic acid (GCA), and glycoursodeoxycholic acid (GUDCA) have been shown to significantly inhibit tube formation and endothelial cell proliferation and migration (Kundu et al., 2017; Warden and Brantley, 2021). *In vivo* experiments, ursodeoxycholic acid (UDCA) suppressed pathological retinal vasodilation by reducing the expression of inflammatory factors and normalizing VEGF-STAT3 signaling (Thounaojam et al., 2020).

**FIGURE 3:**
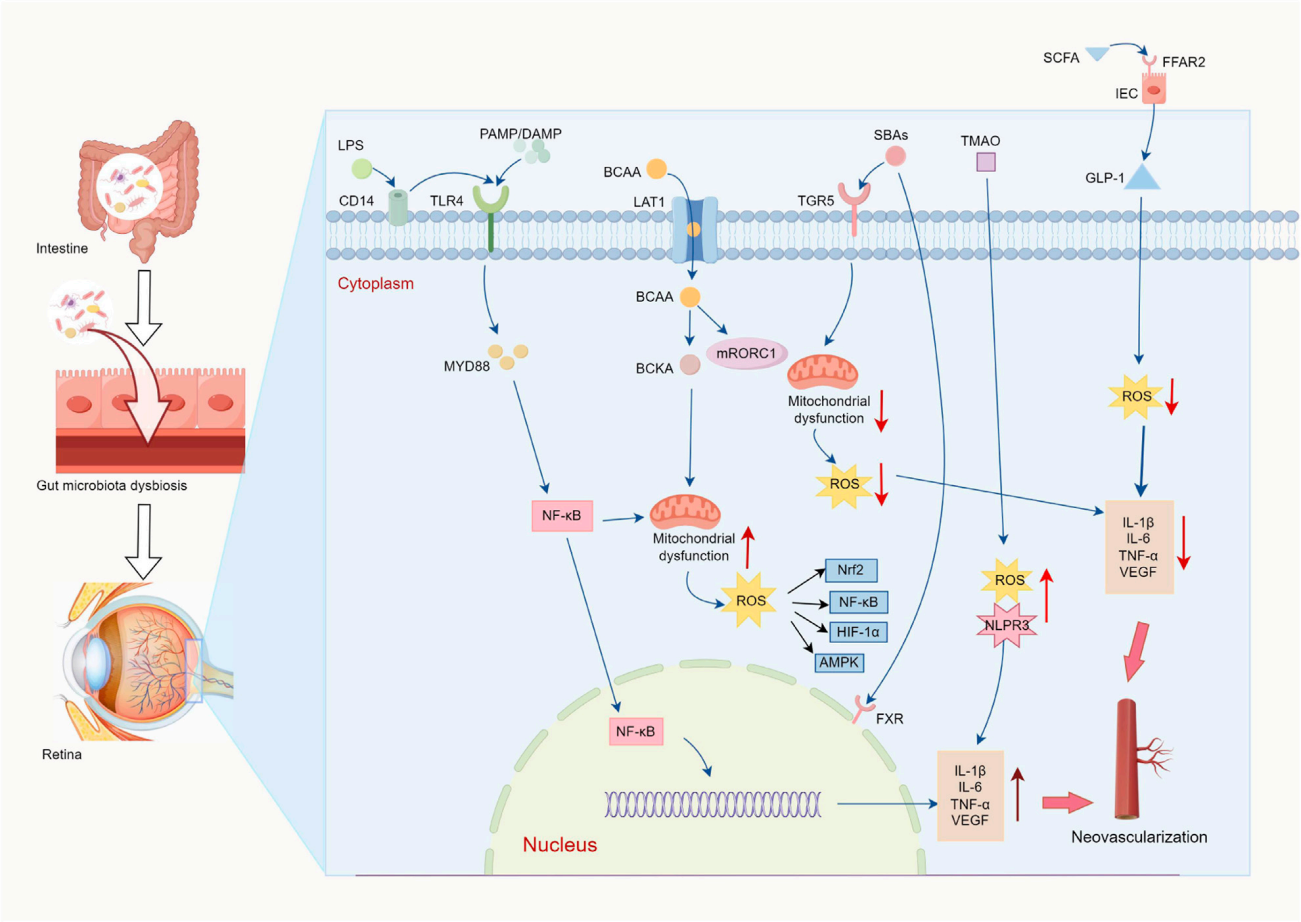
Schematic diagram of the “gut-blood-retina” regulatory mechanism. Poor microecological intestinal regulation of disease status (pathogenic bacteria↑/probiotic bacteria↓) leads to a weakening of intestinal barrier function, and intestinal hyperpermeability induces the entry of harmful metabolites, such as LPS and BCAA, into the bloodstream through the portal vein, and reduces beneficial metabolites, such as SCFAs and BAs. LPS regulates the expression of VEGF and pro-inflammatory factors (TNF-α, IL-6) by signaling TLR4/NF-κB. BCAAs enter retinal cells *via* the amino acid transporter (LAT1) in retinal cells, activating the mTORC1 pathway to stimulate insulin resistance, inflammation, ROS production and mitochondrial dysfunction, resulting in reduced cell activity. SCFAs increase GLP-1 secretion through FFARs and inhibit ROS-induced pro-inflammatory factors and VEGF expression. BAs improve mitochondrial function through TGR5/FXR receptor function, as well as inhibition of inflammatory responses and anti-angiogenesis. While the above gut microbial and metabolite disorders ultimately lead to BRB disruption, vascular leakage and neovascularization, DP address this process through a triple mechanism: (1) modification of GM composition and reduction of LPS and BCAAs biosynthesis; (2) improve intestinal barrier (tight junction proteins↑, sIgA↑) and prevent enterotoxin penetration into the bloodstream; (3) metabolic remodeling: promoting the production of SCFAs (FFAR2/3↑), activation of the SCFA-GPCR41/43 pathway to improve endothelial function; Positive regulation of the catabolic BCAAs enzymes (BCATs, BCKDH), inhibiting the over-activation of mTORC1; Regulate BAs metabolism (TGR5↑), stimulate polarization of M2 macrophages and reduce phagocytic activity and inflammatory response. In addition, DP can regulate endothelial energy metabolism *via* the fatty acid/VEGF-B/VEGFR1 axis, thus providing a new target for anti-angiogenic treatment (↑increase; ↓decrease).

In this context, DP, as a microecological regulator of the gut, exhibits multidimensional mediation potential (Liu et al., 2023; Wu et al., 2023; Zhang and Mo, 2023). DP can optimize the composition of GM by increasing the relative abundance of beneficial bacteria (e.g., *Bifidobacterium*, *Lactobacillus*) and reducing the number of harmful bacteria (e.g., *Escherichia coli*, *Staphylococcus*) (Chen X. et al., 2023; Wu et al., 2023), which maintains the structural balance of GM and prevents overproduction of harmful metabolites. DP has been shown to improve the protective function of the intestine (Xie et al., 2019). By regulating the expression of the connection proteins of the intestinal mucosa (e.g., Occludin and ZO-1) and increasing the secretion of sIgA, β-defensin and mucin-2, it has been able to improve the morphology and structure of the intestinal mucosa and increase the activity of immune cells in the intestinal tract and blood circulation, thus increasing intestinal barrier function and systemic immune response. Meanwhile, this study also found that it reduced the abundance of the *Proteobacteria* derived from LPS and also reduced the abundance of the *Firmicutes* and *Bacteroidetes* phyla. Therefore, DP can inhibit LPS production and LPS transmission of TLR4-NF-κB signaling pathways (Wang et al., 2020), reduce the release of pro-inflammatory and pro-angiogenic factors, and reduce intestinal hyperpermeability, thereby reducing retinal damage caused by pathogenic bacteria and the release of harmful toxins. It is interesting to note that the increase in *Firmicutes* episodes is associated with high intestinal permeability, chronic low-grade inflammation and has been shown to increase neovascularization (Zinkernagel et al., 2017). DP has been shown to alter BCAA metabolism by suppressing genomic expression associated with BCAA biosynthesis and increasing regulation of the catabolic BCAAs enzymes (BCATs, BCKDH) (Chen H. et al., 2021). This reduces the systemic accumulation of BCAAs, thereby mitigating the expression of mTOR-mediated inflammatory factors and VEGF activation. At the same time, polysaccharides attenuate ROS generation while maintaining mitochondrial integrity while maintaining cellular stability. In addition, DP increases microbial populations of SCFAs-producing taxa (e.g., *Lactobacillus*), stimulates the biosynthesis of SCFAs and improves receptor signaling pathways (FFAR2/FFAR3). These microbial metabolites act as metabolic substrates for intestinal epithelial cells, combat mitochondrial damage caused by ROS, and exhibit a variety of bioactivities, including antioxidant, anti-inflammatory and immunoregulatory properties (Liu et al., 2023; Zeng et al., 2024). Another study has demonstrated that DP enhances vascular endothelial functionality by activating the gut-vascular SCFA-GPCR43/41 signaling axis (Li Y. et al., 2021). DP can restore metabolic homeostasis of BAs in the systemic circulation, thereby improving fat absorption and potentiating insulin secretion under pathological conditions (Yang J. et al., 2020). From a mechanistic point of view, these polysaccharides suppress retinopathic progression *via* dual pathways: (1) facilitating interactions of BAs-TGR5 on macrophage membranes to induce anti-inflammatory polarization of M2 and reduce phagocytic activity and pro-inflammatory cytokine secretion; (2) upregulating interleukin-10 (IL-10) expression to antagonize angiogenesis-promoting microenvironments (Feng et al., 2019; Liu H. et al., 2021). Interestingly, BAs can transport fatty acids to the apical membrane of intestinal epithelial cells and absorb them into the bloodstream, after which they are absorbed by VEGF-B controlled vascular endothelium mediated by vascular fatty acid transport proteins, thereby participating in endothelial energy metabolism (Hagberg et al., 2010). This process may be related to VEGF-B/VEGFR1 signaling to regulate the expression of fatty acid transporter proteins in an endothelium-mediated manner, which may suggest that the future therapeutic potential of DP for anti-angiogenesis could be explored through the fatty acid/VEGF-B/VEGFR1 pathway (Figure 3).

### 4.6 The structure-activity relationship between *Dendrobium* polysaccharides and anti-angiogenesis

Polysaccharides are complex macromolecules that are widespread and commonly found in animals and plants. The biological activity of these polymers, which are composed of several glycosidically linked monosaccharide units, is closely related to their structural properties, including monosaccharide composition, molecular weight, types of glycosidic bonds and structural modifications (He et al., 2018; Bu et al., 2019). DP is no exception, and their antiangiogenic activity is closely related to the complex features of their molecular structure, particularly their monosaccharide composition, sulfate modifications and molecular weight. As shown in Table 1, the main monosaccharides of DP are Glcp and Manp, which are also frequently found in polysaccharides from various plant sources. These monosaccharides are alternatively linked by (1→4)-α/β-D-Manp or (1→4)-α/β-D-Glcp, forming the basis of glucan or heteropolysaccharides (Li Y. et al., 2023). Although the composition of polysaccharide monosaccharides varies among *Dendrobium* species, some common structural features are associated with their antiangiogenic properties. In particular, the addition of modifying groups, such as sulfate and acetyl groups at the C-2, C-3, or C-6 positions of monosaccharide residues, is known to significantly increase the antiangiogenic properties of DP.

Several natural polysaccharides containing sulfate groups have been shown to have a wide range of biological effects, including anticoagulant, antiviral, and anticancer properties (Chen et al., 2010). In recent years, the addition of sulfate groups to specific regions of DP has been used to alter their molecular conformation and increase their binding affinity to targets. This modification has attracted much attention due to its unique structural patterns and potent anti-angiogenic activity. The data are summarized in Table 2. For example, JCS1S2 from *Dendrobium nobile Lind* (sulfate group at C-6 position, sulfation degree 1.74, Mw 56.2 kDa), whose modification by sulfation significantly increased its affinity for VEGF (*K*_*D*_ = 4.82 × 10^−9^) and had potent antiangiogenic effects *in vitro* and *ex vivo* by competitively inhibiting VEGF-VEGFR2 signaling (p < 0.001) (Wang H. Y. et al., 2019). In the OIR model, it reduced the area of neovascularization *via* the TLR4/p-NF-κB/VEGF pathway by about 60%, and significantly reduced the retinal vessel non-perfusion area (p < 0.05) and Müller cell activation (Bai et al., 2025). This is crucial for the treatment of retinal neovascularization (RNV) and indicates that JCS1S2 has therapeutic potential to inhibit RNV formation and is associated with downregulation of VEGF production and inflammatory events. High levels of sulfation are known to significantly increase the binding affinity of polysaccharides to their targets and directly influence their anti-angiogenic effects (Chen et al., 2012). Similarly, sulfated Dendrobium officinale Kimura & Migo polysaccharide S32S (degree of sulfation 0.9, Mw 54 kDa) inhibits endothelial cell tube formation at a low concentration of 0.29 μM and stops tube formation completely at 0.58 μM (Yue et al., 2017). S32S shows dose-dependent antiangiogenic effects without significant cytotoxicity, which is superior to the unmodified natural polysaccharide S32 (up to 13.51 μM). Although polysaccharides from different *Dendrobium* sources (e.g., *D. nobile Lindl* JCS1S2 and Dendrobium officinale Kimura & Migo S32S) differ in monosaccharide composition (Table 1), the main chain in both consists of (1→4)-β-D-Manp and (1→4)-α-D-Glcp with sulfation sites concentrated at the C-6 position. This structural homology may be the common basis for their anti-RNV activity. The polysaccharides DHPD1 (sulfation 1.473, Mw 3.2 kDa) (Qian et al., 2014) and DHPD2 (sulfation 0.94, Mw 8,090 kDa) (Li et al., 2014) are different sources of the above polysaccharides, but both show higher antiglycation activity after sulfation at the C-2 or C-6 position. They are expected to alleviate the progression of diabetic vascular complications by inhibiting the protein glycation process. These studies suggest that the synergistic effects of sulfation modification and specific glycosidic linkages, rather than the plant origin itself, increase the aqueous solubility of polysaccharides and alter their spatial conformation, which may be critical for activity against RNV.

**TABLE 2:** Structure-anti-angiogenic activity association of sulfated polysaccharides.

*Dendrobium* spp.	Sulfated polysaccharide name	Molecular weight	Main chain	Sulfated modification	Antiangiogenic activity (inhibition rate)	References
*Dendrobium nobile* Lind	JCS1S2	56.2 kDa	1,4-β-D-Manp, 1,4-α-D-Glcp	C-6 position, DS 1.74	60% (20 μg/μL, OIR model)	Bai et al. (2025)
*Dendrobium officinale* Kimura & Migo	S32S	54 kDa	1,4-β-D-Manp, 1,4-α-D-Glcp	C-6 position, DS 0.9	100% (0.58 μM, *In vitro* tube-forming assay	Yue et al. (2017)

Further structure-activity studies showed a close correlation between the molecular weight of DP and its biological activity. Studies have shown that low molecular weight DP possess potent antioxidant activity (Wang et al., 2022), such as DOP 80 and DOP 70 from Dendrobium officinale Kimura & Migo (Fan et al., 2018; Liu Y. et al., 2021), whereas higher molecular weight polysaccharides exhibit stronger antitumor and immunomodulatory activity (Feng et al., 2020). This phenomenon may be closely related to the structural complexity of polysaccharide molecular chains, glycosyl patterns and types of glycosidic linkages. For example, lower molecular weight polysaccharides of *Dendrobium* often contain higher amounts of arabinose and rhamnose, which are closely related to their higher antioxidant properties (Fan et al., 2018). Conversely, higher molecular weight polysaccharides, due to their longer molecular chains and more complex conformation, may bind more efficiently to angiogenesis-related targets such as VEGF, thereby inhibiting the formation of new blood vessels.

Moreover, acetylation modification of DP significantly affects their bioactivity, and the higher the degree of substitution, the higher the antioxidant activity (Wang et al., 2022). Studies have shown that DP with O-acetyl groups have higher immunomodulatory and anticancer activity. The linear chain structure formed by (1→6)-Glcp and (1→4)-Manp is probably the structural basis for this activity, and their antiangiogenic activity is closely related to their molecular weight (He et al., 2024). For example, polysaccharides isolated from Dendrobium huoshanense Z.Z.Tang & S.J.Cheng containing O-acetyl groups showed significantly greater inhibition of angiogenesis than their non-acetylated counterparts. Moreover, the antiangiogenic activity of these O-acetylated polysaccharides was proportional to their molecular weight, further supporting that the degree of acetylation and molecular weight play a role in modulating the antiangiogenic potential of polysaccharides (Liu H. et al., 2021).

In general, the antiangiogenic activity of DP is not only closely related to monosaccharide composition and skeletal structure, but also depends on factors such as the degree of chemical modification, including sulfation and acetylation, molecular weight and glycosylation pattern. By modifying their structural properties, notably through sulfation and acetylation, the antiangiogenic activity of these polysaccharides can be significantly enhanced, reinforcing their potential as anti-RNV agents. Although the exact relationship between the structural properties of DP and their antiangiogenic activity is still under investigation, the available data indicate that factors such as structural homology, the addition of modifying groups and molecular mass play an important role in their anti-RNV activity. Future studies should therefore continue to investigate the relationship between the structure and activity of DP, and optimize their biological activity through chemical modifications that could ultimately enhance their use in ophthalmology.

## 5 Discussion

RNV is a challenging problem in many clinical fundus diseases, such as AMD and DR. Developing strategies to inhibit RNV has become a major focus of ophthalmic pharmacology, but the number of effective, safe and long-term treatment options is still limited. RNV formation is a complex process involving multiple mechanisms, with the VEGF signaling pathway, which controls endothelial cell proliferation, migration and lumen formation, playing a central role. In addition, factors such as disruption of the BRB, inflammation, oxidative stress and endothelial cell damage contribute to RNV formation. This process is accompanied by the loss of pericytes, the formation of non-perfusion areas and vascular leakage, ultimately leading to a loss of vascular stability and the formation of pathological blood vessels. In response to these mechanisms, DP, especially sulfated polysaccharides, have been regarded as potent candidates for anti-RNV therapy due to their low cytotoxicity and significant antiangiogenic activity, as well as their unique advantages in inhibiting the formation of RNV at multiple targets. For example,: (1) Direct binding to VEGF: SPR analysis shows that JCS1S2’s affinity for VEGF (*K*_*D*_ = 4.82 × 10^−9^) is much higher than that of VEGFR2 (*K*_*D*_ = 1.50 × 10^−7^) and higher than that of some clinical anti-VEGF drugs (e.g., bevacizumab with *K*_*D*_ = 58 pM) (Papadopoulos et al., 2012); (2) Remarkable *in vivo* efficacy: intravitreal injection of JCS1S2 reduces VEGF-A expression by about 60% and reduces the area of neovascularization by about 50% in the OIR model (Bai et al., 2025); (3) High safety: no toxicity for HUVECs at concentrations of 50–800 μg/mL (Li B. et al., 2021). Despite the growing evidence of the anti-RNV properties of DP, several challenges remain in this area of research.

DP derived from various plant species exhibits differences in extraction methods, molecular weight, glycosidic linkage types, and monosaccharide composition. These variations constitute a primary reason for the inconsistency observed in their bioactivities, posing significant challenges to elucidating their structure-activity relationships. The key limitations include (1) Debate on critical functional groups: Sulfation is widely recognized as a key modification enhancing anti-angiogenic activity. However, quantitative consensus regarding the impact of sulfate group position (C-2 or C-6) and degree of substitution (DS, ranging from 0.9 to 1.74) on bioactivity remains elusive across different studies. However, the influence of the sulphate position (C-2 or C-6) and the degree of substitution (0.9–1.74) on activity is inconsistent across studies, making standardized comparisons of biological activity difficult (Li et al., 2014; Qian et al., 2014; Yue et al., 2017). (2) Impact of structural heterogeneity: While DP from different botanical sources may possess variations in minor monosaccharide constituents or specific glycosidic bond structures, the extent to which these structural differences significantly influence their anti-angiogenic potency is poorly understood. This uncertainty makes it almost impossible to make meaningful comparisons or draw general conclusions based on the “structure-activity relationships” described in the various studies. (3) Molecular weight-activity correlation: High-MW polysaccharides, characterized by extended chain length and complex conformation, potentially exhibit higher VEGF binding efficiency. Conversely, low-MW polysaccharides demonstrate superior capacity to penetrate the BRB, thereby facilitating antioxidant effects and barrier protection (Yue et al., 2017; Wang et al., 2022). (4) Lack of standardized extraction: Different extraction techniques (e.g., ultrasound-assisted, enzymatic, or acid extraction) can induce variations in MW, glycosidic bond conformation, and the degree of functional group modifications. These alterations consequently affect the resulting biological activity profiles. Future studies should therefore systematically evaluate the specific influence of sulfate degree, localization and molecular weight on the antiangiogenic activity of DP using standardized extraction protocols, structure characterization and activity assessment to ensure the reliability of polysaccharide assays.

Although DP demonstrates significant anti-RNV activity in animal models (e.g., OIR rats) and *in vitro* assays (e.g., HUVEC cells), its reliability may decrease because of small sample sizes and the use of a single model and the short treatment duration. Furthermore, the effectiveness on real-life clinical outcomes, like functional outcomes (e.g., visual improvement, vascular leakage reduction), is currently unknown. Otherwise, although DP exhibits nontoxicity *in vitro* and experimental animals, not many studies refer to the long-term toxicity of DP and its clinical use. Most toxicity studies are based on HUVEC cell models, do not investigate effects on other cell types (e.g., retinal pigment epithelial cells, Müller cells, neurons), and do not consider long-term effects. Therefore, future research should focus on comparative studies between different animal species, especially by selecting animal and cell models that mimic human retinal pathology to increase the clinical value of the studies.

DP has shown favorable bioactivity in recent studies, but the problem of their low bioavailability after oral administration due to their high molecular weight and viscosity remains unresolved (Li et al., 2019; Wu et al., 2022). According to the existing hypothesis of the intestinal-gut axis, DP in appropriate concentrations modulates the integrity of the intestinal barrier, alters the composition of the gut microbiota, and restores the metabolic homeostasis of the intestinal environment and systemic circulation, thus exerting bioactivity indirectly. Studies have shown that in the presence of polysaccharide utilization loci (PULs), DP may be degraded by the phylum *Bacteroidetes* and the genus *Prevotella* into small molecules such as manno-oligosaccharides, mannose, and SCFAs, thereby indirectly affecting the retinal microenvironment (Li et al., 2019; Lei et al., 2022; Zhao et al., 2023). However, these studies lack data on the retinal degradation of DP, and it remains unclear whether it crosses the BRB. Consequently, future investigations should integrate metagenomics and metabolomics to determine how gut microbiota-derived metabolites of DP (e.g., SCFAs, oligosaccharides) regulate the retinal microenvironment through circulatory or neural signaling. Furthermore, highly sensitive tracking techniques using isotope labeling and imaging would be required to look at the possibility of oral DP and/or its key metabolites reaching the retina and their concentrations in the upcoming research. In terms of clinical applications, based on standardized DP, the relationship between dose and effect, the impact of individual microbiota variations on efficacy, and the consistency of efficacy across different species, should be validated through more *in vivo* and clinical trials.

Furthermore, the optimal clinical delivery route for DP needs to be further investigated. While oral administration offers enhanced patient compliance and the potential to indirectly modulate the retinal microenvironment *via* the gut-retina axis, this route is significantly limited by low bioavailability and a strong dependence on gut microbiota composition and activity. Intravitreal injection or ocular implants can bypass the BRB, delivering drugs directly to the site of pathology with controlled dosages and rapid onset of action. This approach addresses the issue of poor bioavailability. However, these methods are invasive and may induce immune responses or cause vitreous opacities due to the large molecular size of polysaccharides. Additionally, no data on the ocular tolerance of DP are available at present, necessitating further evaluation of dose-dependent toxicity and long-term safety. Designing nanoparticles capable of crossing the BRB, such as liposomes, exosomes, or chitosan nanoparticles, encapsulating large molecular polysaccharides, can prevent degradation in the gastrointestinal tract, enhance blood concentration, and facilitate delivery to the retina *via* systemic or local administration. This approach may enable low-toxicity and long-lasting therapeutic effects of polysaccharides. However, the long-term toxicity of nanoparticle carriers has yet to be evaluated, and the production process is complex. Additionally, the *in vivo* metabolic pathways remain unclear, necessitating careful assessment of the safety of the carriers. Structural modifications, such as reducing molecular weight, increasing water solubility, or altering glycosidic bond structures, can enhance the transmembrane absorption of DP. However, these modifications may alter the natural conformation of the polysaccharides. And, there is currently a lack of systematic studies to assess the retention of activity and the potential new toxicity of the modified products. Therefore, a balance between structural modification and safety must be maintained. Furthermore, co-administration of probiotics and prebiotics as a pretreatment may help improve the bioavailability of DP. Tailoring dietary plans based on individual gut microbiomes could further optimize the biological activity of DP.

In summary, DP has been regarded as a promising therapeutic candidate for the treatment of retinal diseases due to its unique polypharmacological properties: inhibition of angiogenesis, immunomodulatory and ROS-reducing ability, combined with low cytotoxicity in preclinical studies. The follow-up study should cover the existing gaps in the study area. For instance, the regulatory mechanisms of DP in VEGF-independent metabolic pathways should be further investigated. The human retinal organoid model should be created, and the combination of tracking techniques and multiomic studies should be used to investigate the accumulation and metabolism of DP in the retina. Computer-aided design should be used to predict the binding conformations of DP-VEGF, and standardized methods of extraction, purification, and specific structural characterization should be used for accurate structural optimization. Phase I clinical trials should be directed at finding the advantages and disadvantages of the different routes of administration prescribed (oral, injection, nanoparticle carriers, *etc.*) and their long-term toxicity. The development and optimization of combined oral and ocular strategies should be investigated to improve clinical applicability and ultimately ensure sustainable treatment of retinal diseases.
